# Enhancing Visible-Light Photocatalysis with Pd(II) Porphyrin-Based TiO_2_ Hybrid Nanomaterials: Preparation, Characterization, ROS Generation, and Photocatalytic Activity

**DOI:** 10.3390/molecules28237819

**Published:** 2023-11-28

**Authors:** Dawid Malec, Marta Warszyńska, Paweł Repetowski, Anton Siomchen, Janusz M. Dąbrowski

**Affiliations:** 1 Faculty of Chemistry, Jagiellonian University, 30-387 Kraków, Poland; dawiid.malec@student.uj.edu.pl (D.M.); m.warszynska@doctoral.uj.edu.pl (M.W.); pawel.repetowski@doctoral.uj.edu.pl (P.R.); anton.siomchen@student.uj.edu.pl (A.S.); 2 Doctoral School of Exact and Natural Sciences, Jagiellonian University, 30-348 Kraków, Poland

**Keywords:** metal complexes, porphyrin, reactive oxygen species (ROS), singlet oxygen, titanium dioxide (TiO_2_)

## Abstract

Novel hybrid TiO_2_-based materials were obtained by adsorption of two different porphyrins on the surface of nanoparticles—commercially available 5,10,15,20-tetrakis(4-sulfonatophenyl)porphyrin (TPPS) and properly modified metalloporphyrin—5,10,15,20-tetrakis(2,6-difluoro-3-sulfophenyl)porphyrin palladium(II) (PdF_2_POH). The immobilization of porphyrins on the surface of TiO_2_ was possible due to the presence of sulfonyl groups. To further elevate the adsorption of porphyrin, an anchoring linker—4-hydroxybenzoic acid (PHBA)—was used. The synthesis of hybrid materials was proven by electronic absorption spectroscopy, dynamic light scattering (DLS), and photoelectrochemistry. Results prove the successful photosensitization of TiO_2_ to visible light by both porphyrins. However, the presence of the palladium ion in the modifier structure played a key role in strong adsorption, enhanced charge separation, and thus effective photosensitization. The incorporation of halogenated metalloporphyrins into TiO_2_ facilitates the enhancement of the comprehensive characteristics of the investigated materials and enables the evaluation of their performance under visible light. The effectiveness of reactive oxygen species (ROS) generation was also determined. Porphyrin-based materials with the addition of PHBA seemed to generate ROS more effectively than other composites. Interestingly, modifications influenced the generation of singlet oxygen for TPPS but not hydroxyl radical, in contrast to PdF_2_POH, where singlet oxygen generation was not influenced but hydroxyl radical generation was increased. Palladium (II) porphyrin-modified materials were characterized by higher photostability than TPPS-based nanostructures, as TPPS@PHBA-P25 materials showed the highest singlet oxygen generation and may be oxidized during light exposure. Photocatalytic activity tests with two model pollutants—methylene blue (MB) and the opioid drug tramadol (TRML)—confirmed the light dose-dependent degradation of those two compounds, especially PdF_2_POH@P25, which led to the virtually complete degradation of MB.

## 1. Introduction

The generation of reactive oxygen species (ROS) is an important aspect of not only biological and biochemical processes but also environmental processes [1]. Moreover, ROS play a key role in other applications such as photodynamic therapy of cancer (PDT) [2], photodynamic inactivation of microorganisms (PDI) [3], as well as photodegradation of dyes, drugs, polymers, and various organic compounds. In the ever-expanding field of heterogenous photocatalysis, there is great interest in developing new hybrid materials capable of efficiently activating small molecules [4]. Porphyrins, as the most extensively studied tetrapyrrolic compounds, are well-known photogenerators of ROS [5]. They strongly absorb light from the visible range, which is associated with π–π* electron transitions, and the energy thereby absorbed is converted into various photophysical processes and photochemical reactions. Therefore, research on porphyrins includes not only photomedicine but also optoelectronics, photocatalysis, and solar energy conversion [6,7].

Porphyrins can coordinate metal ions, which significantly modify their spectroscopic, photophysical, and photochemical properties. Incorporation of various metal ions into the center of the compound also affects their stability, hydrophilicity, and tendency to aggregation, which, in sum, affects the efficiency of photo-oxidation processes [8,9,10]. Modification of the structure of porphyrins with substituents also has a significant impact on their solubility, stability, and photochemical properties [11,12]. Previous studies have shown that halogenated tetrapyrroles exhibit much higher spin-orbit coupling constants than unmodified analogues, which increases the quantum yield of the intersystem crossing (ISC) [13]. Modification by the attachment of thioester or sulfonamide groups, on the other hand, introduces a steric hinge that increases the overall stability of the molecule [14].

In recent years, metal oxide nanoparticles have garnered widespread interest from researchers in various fields (biomedicine, bioimaging, biosensors, and catalysis) due to their unique physical and chemical properties [15,16,17,18,19]. TiO_2_—an n-type semiconductor—is one of the most intensively investigated catalysts, characterized by low toxicity accompanied by its high photochemical and thermal stability and strong oxidizing capability [20,21,22,23]. For this reason, it is widely used in biomedical research but also in environmental applications, e.g., as a catalyst in air or water purification [24,25,26,27,28]. It is also used in self-cleaning surfaces, in CO_2_ reduction [29,30], in H_2_ generation [31], and in the catalytic decomposition of harmful organic compounds [32,33,34,35]. The development of nanomaterials has allowed for the improvement of their properties, including the increase in the absorbing material’s band gap in the ultraviolet range. However, there is a large bandgap of TiO_2_ (E_g_ = 3.2 eV for anatase, E_g_ = 3.0 eV for rutile) that determines its limited (ca. 5%) ability to harvest natural sunlight [36,37,38]. Those disadvantages can be overcome with different modifications such as metal/non-metal doping, co-doping, and sensitization with organic dyes [36,37,38,39,40,41,42]. The latter, however, does not function as photocatalysts but will contribute to the hybrid material by leveraging their unique properties. The photosensitization of the materials is based on the sensitizer adsorption to the semiconductor surface and the absorption of photons of an appropriate wavelength, which transition into an excited state. The primary purpose of the sensitizer is to overcome the inherent limitations of inorganic semiconductors [43]. In this paper, we will focus on the photosensitization of TiO_2_ with properly designed porphyrin derivatives.

One of the most important features of titanium (IV) oxide is its importance in advanced oxidation processes (AOP), which are related to efficient ROS generation [44]. During the activation of TiO_2_ particles with light from the UV range, electrons and holes in the conduction and valence bands are generated [45,46]. The generated charges take part in the oxidation and reduction reactions that occur on the TiO_2_ surface [47]. In the case of ROS generation, electrons and holes react with water and O_2_ adsorbed on the surface, resulting in the formation of hydroxyl radicals and superoxide ion radicals [48]. This phenomenon can be used, among others, in the photodynamic inactivation of microorganisms, as ROS generated in this way react with biological structures such as lipids, proteins, or DNA, leading to their damage [49]. The best photocatalytic activity of TiO_2_ is obtained for anatase or a mixture of anatase and rutile [50]. Titanium (IV) oxide is distinguished from other semiconductors by the long lifetime of the generated hole-electron pairs, which further increases the efficiency of the subsequent photochemical reactions [51]. In addition, substances with anchor groups such as -OH, -COOH, and -SO_3_H, phosphates, and metal ions (such as Zn^2+^ and Pd^2+^) bind well to its surface, further stabilizing the semiconductor-modifier system [49].

Nowadays, numerous organic pollutants from industrial sources, such as synthetic dyes, pesticides, pharmaceuticals, and solvents, pose a serious threat to both the environment and human well-being, especially through water resources. Methylene blue (MB), an artificial cationic dye, is widely used in various industries such as textiles, cosmetics, leather, and pulp, making it a major contributor to wastewater pollution. It is present not only in wastewater from its industry but also in wastewater from other sectors. Human overexposure to MB has been linked to health issues, including tissue degradation, nausea, shock, and an irregular heartbeat. Consequently, a few techniques have been developed to eliminate these contaminants from water and wastewater to limit their impact on human health and the environment [52,53]. Among various approaches, photocatalytic applications have gained considerable popularity to address these challenges. Metal oxide nanoparticles are widely used in the photocatalytic degradation of these organic pollutant dyes, providing an effective means of remediation. TiO_2_ is the most suitable photocatalytic compound due to its superior photocatalytic activity over a wide temperature and pH range [54,55]. Other hybrid materials can also be used for photocatalytic degradation of various pollutants, such as the perylene imide/Bi_2_WO_6_ hybrid photocatalyst used for the degradation of Bisphenol A. The polymeric compound facilitates the charge separation of carriers, increasing the activity of Bi_2_WO_6_ [56]. MB is extensively used as a model pollutant in photocatalytic tests, and the process of its degradation is well documented. TiO_2_-based photocatalysis may lead to the successful oxidation of dyes, resulting in the mineralization of carbon, sulfur, and nitrogen into CO_2_, SO_4_^2−^, NH4^+^, and NO_3_^−^ [57]. However, MB as a model pollutant can pose some problems. It has been observed that during irradiation with UV radiation, MB molecules undergo desorption from the surface of the catalyst. The initial concentration of the dye has an impact on the rate of the photodegradation reaction. High concentrations of MB can even suppress the degradation process [58]. The photodegradation process of MB is also significantly slower under visible than UV irradiation [59].

In our study, we focused on understanding the physicochemical changes in TiO_2_ (P25) after anchoring porphyrins on the surface of nanoparticles and the effects of palladium ion incorporation on the photochemistry of hybrid materials. For our research, we used two different porphyrins—commercially available non-metallic porphyrin—5,10,15,20-tetrakis(4-sulfonatophenyl)porphyrin (TPPS) and synthesized porphyrin complexed with Pd^2+^-5,10,15,20-tetrakis(2,6-difluoro-3-sulfophenyl)porphyrin palladium (II) (PdF_2_POH). These porphyrins were anchored with sulfonic groups to the titanium (IV) oxide surface. Palladium (II) itself also helps to anchor the porphyrin to the TiO_2_ surface. In addition, the effect of 4-hydroxybenzoic acid (PHBA) as a linker to increase the amount of attached porphyrin to the TiO_2_ surface was also studied. Introducing PHBA on the P25 surface could potentially lead to increased stability of the resulting composite, thus increasing photocatalytic activity [60]. Moreover, the presence of palladium ions in porphyrin-based materials resulted in increased photoinduced charge separation compared to metal-free or zinc derivatives [61,62]. To confirm the adsorption of porphyrins on the P25 surface, UV-Vis electronic absorption measurements were conducted, along with nanoparticle size evaluation and ζ-potential analysis by DLS. We investigated the photosensitization of TiO_2_-based materials by photocurrent measurements and ROS generation changes with the use of two probes: singlet oxygen sensor green (SOSG), which is a selective ^1^O_2_ probe, and 3′-(*p*-aminophenyl) fluorescein (APF), selective for hydroxyl radical. Finally, we investigated the photocatalytic properties of the obtained materials with two model pollutants—dye methylene blue and an example of an opioid drug—tramadol hydrochloride.

## 2. Results

### 2.1. Spectroscopic Characterization of Photosensitizers

Our research starting point was porphyrins such as 5,10,15,20-tetrakis(4-sulfonatophenyl)porphyrin—TPPS or halogenated 5,10,15,20-tetrakis(2,6-difluoro-3-sulfophenyl)porphyrin—F_2_POH (previously described in some of our articles) [49]. However, metal-free porphyrins tend to aggregate in aqueous solutions [63]. By introducing sulfonyl groups, the solubility of the porphyrin can be controlled, leading to changes in spectroscopic properties and the possibility of J-type aggregation, depending on concentration and pH value [64]. To prevent this undesirable process, which can hinder the generation of ROS and reduce activity, we utilized porphyrin derivatives with electron-withdrawing groups. Specifically, the incorporation of fluorine atoms at the ortho-position of the phenyl rings significantly decreases aggregation, influences the redox properties, and stabilizes the porphyrin structure against (photo)oxidation. Moreover, in this work, we incorporated the Pd^2+^ ion into the halogenated porphyrin structure, which increases the triplet quantum yield and, therefore, enhances the generation of reactive oxygen species. The synthesis of porphyrins was conducted according to the protocols published before [65].

**Figure 1:**
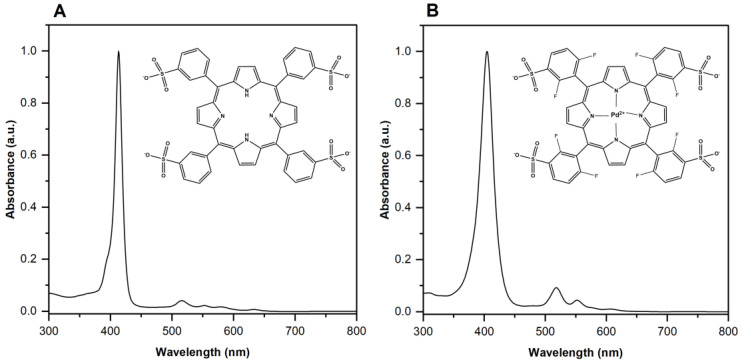
Chemical structure and electronic absorption spectra of 5,10,15,20-tetrakis(4-sulfonatophenyl)porphyrin (TPPS) (**A**) and 5,10,15,20-tetrakis(2,6-difluoro-3-sulfophenyl)porphyrin palladium(II) (PdF_2_POH) (**B**).

The ground-state electronic absorption spectra of TPPS and PdF_2_POH were recorded at room temperature in Tris buffer (pH = 7.4) and are presented along with their chemical structures in Figure 1. The studied porphyrins exhibit characteristic absorptions in the 500–700 nm range (Q-bands), corresponding to electronic transitions between the S_0_→S_1_ states. The addition of Pd^2+^ into the porphyrin ring resulted in the broadening of the Soret band and a slight hypsochromic shift in the absorption maximum (for TPPS λ_max_ = 413 nm, for PdF_2_POH λ_max_ = 404 nm). This is caused by the back-donation from Pd(II) *d*-orbitals to the porphyrin’s empty π*-orbitals, thus raising the energy gap of the electronic transition. This insertion also resulted in a reduction in the number of Q-bands caused by the change in porphyrin symmetry (D_2h_→D_4h_). The intense absorption in the blue range (Soret band) for both TPPS and PdF_2_POH enables TiO_2_ sensitization to the visible region of the spectrum. In addition, the adsorption of porphyrins on the P25 surface should increase charge separation and result in an enhanced amount of photogenerated ROS.

### 2.2. Adsorption of (Metallo) Porphyrins on TiO_2_ and Their Spectroscopic Characteristics

**Figure 2:**
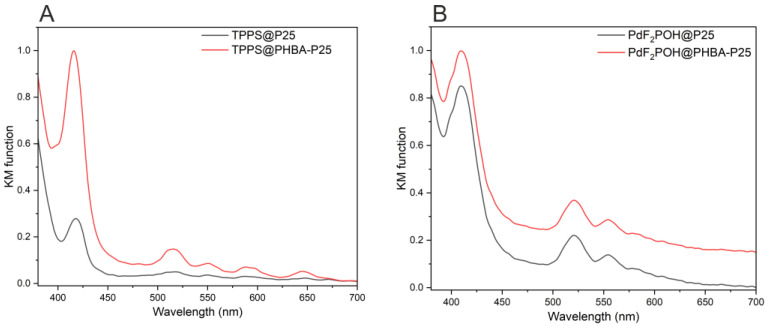
Diffuse reflectance spectra in the representation of the Kubelka–Munk function of TPPS-based materials—TPPS@P25 and TPPS@PHBA-P25 (**A**) and PdF_2_POH-based materials—PdF_2_POH@P25 and PdF_2_POH@PHBA-P25 (**B**).

TiO_2_ (P25) is a white powder characterized by limited absorption of visible light. Thus, we synthesized P25-based materials immobilized with two photosensitizers—TPPS and PdF_2_POH. We designed two series of materials—porphyrins on TiO_2_ and porphyrins on TiO_2_ impregnated with 4-hydroxybenzoic acid (PHBA). The materials were obtained after porphyrin solution sonification and overnight shaking with P25 powder and, optionally, with PHBA. After the adsorption of porphyrins, the powder changed its color from white to beige. The diffuse reflectance spectra of modified TiO_2_ with TPPS and PdF_2_POH and with the incorporation of PHBA into both porphyrins after Kubelka–Munk function calculation are presented in Figure 2.

Electronic absorption spectra confirm the deposition of porphyrins on the TiO_2_ surface. Bare TiO_2_ shows no absorption bands above 400 nm. Adsorption of porphyrins on P25 resulted in a red shift of the Soret band (420 nm) compared to unmodified porphyrins (Figure 1), which indicates S_0_→S_2_ transitions in all tested compounds, proving a successful synthesis of hybrid TiO_2_ materials. Figure 2 shows absorption bands in the 400–550 nm range for both TPPS and the palladium (II) derivative.

### 2.3. Characterization of the Nanomaterials Size and ζ-Potential

**Figure 3:**
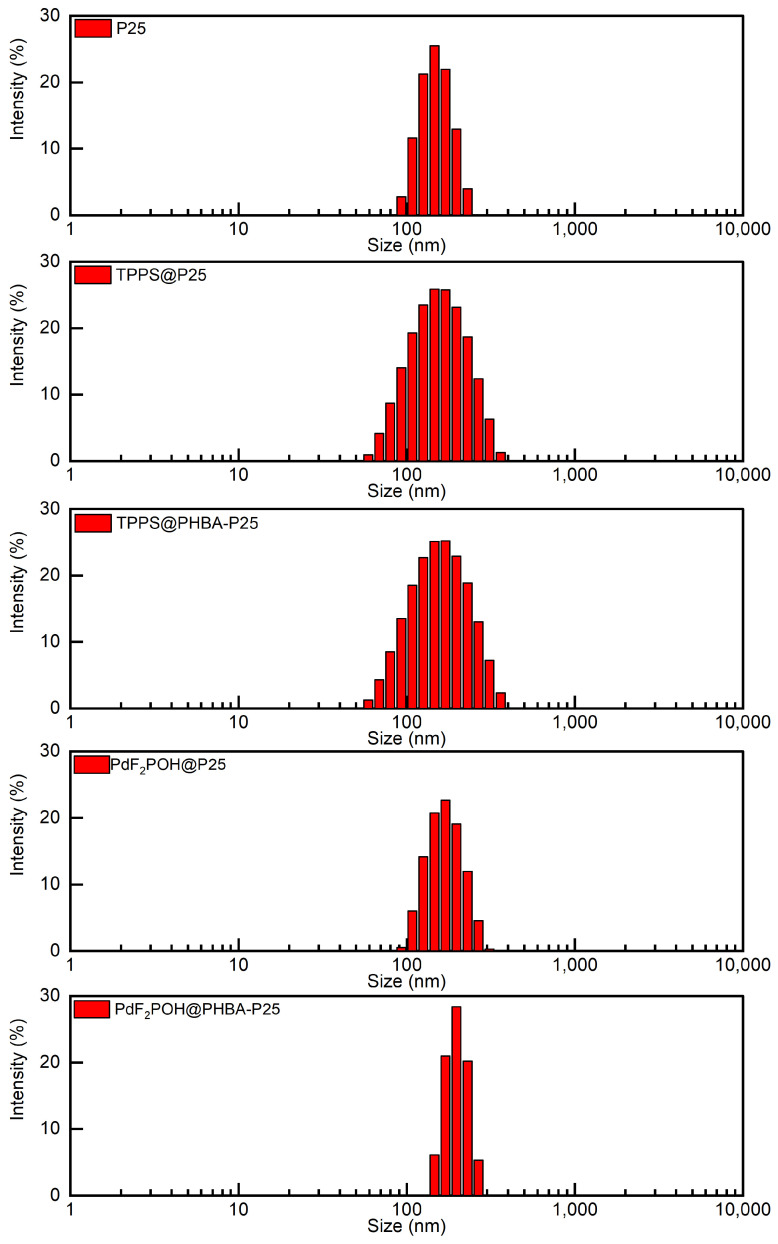
Size distribution of bare P25 and porphyrin-based materials—TPPS@P25, TPPS@PHBA-P25, PdF_2_POH@P25, and PdF_2_POH@PHBA-P25.

To further prove the adsorption of porphyrin on the TiO_2_ surface, the size of the resulting composites and ζ-potential were defined by dynamic light scattering (DLS) measurements (Figure 3, Table 1). The particle size of modified nanomaterials in aqueous solution possesses a homogeneous distribution, with 146 nm for unmodified P25, and 155 nm, 170 nm, and 197 nm, respectively, for TPPS@P25, TPPS@PHBA-P25, PdF_2_POH@P25 and PdF_2_POH@PHBA-P25. The dispersion stability of synthesized hybrid material nanoparticles is characterized by zeta potential (ζ). The obtained values for ζ-potential varied for different formulations. P25 showed a positive potential on the depletion layer (ζ = 21 mV). The lowest ζ-potential was observed for TPPS@P25 and TPPS@PHBA-P25 (ζ = −9 mV and ζ = −33 mV, respectively). The low surface charge of TPPS@P25 can be explained by the structure of the porphyrin itself—negatively charged sulphonyl groups are the source of negative potential. The addition of an acidic linker—PHBA—further lowers the charge of the depletion layer to −33 mV. However, surface modification with palladium derivatives did not result in a ζ-potential decrease, even a slight increase (ζ = 22 mV), which can be due to the fact that positively charged palladium ions reduce the effects of sulphonyl groups. The addition of 4-hydroxybenzoic acid decreased the ζ-potential value to 12 mV. The ζ-potential measurements suggest that TPPS@PHBA-P25 and PdF_2_POH@P25 are the most stable of all the composites obtained and are less likely to aggregate in solutions. All determined values are presented in Table 1.

### 2.4. Photolectrochemical Measurements

TiO_2_ is a semiconductor capable of generating photocurrents within UV irradiation (>400 nm), as it is characterized by a wide bandgap. The absorption of UV photons leads to electron excitation from the valence to the conduction band. This is represented by the current increase.

**Scheme 1:**
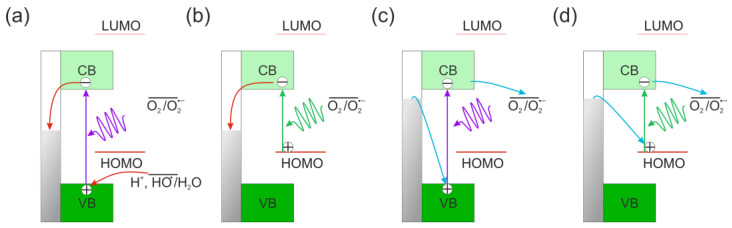
Photocurrent generation mechanism based on [69]: anodic photocurrents originate from positive potential of TiO_2_ excitation (**a**) or excitation of the dye (**b**), cathodic photocurrents are generated upon excitation of TiO_2_ at potentials with negative values (**c**) or by the excitation of complex between the dye and the surface of the material (**d**). The vertical bar indicates the value of photoelectrode potential, and the arrows indicate the transition of electrons from the valence or conduction bands.

**Figure 4:**
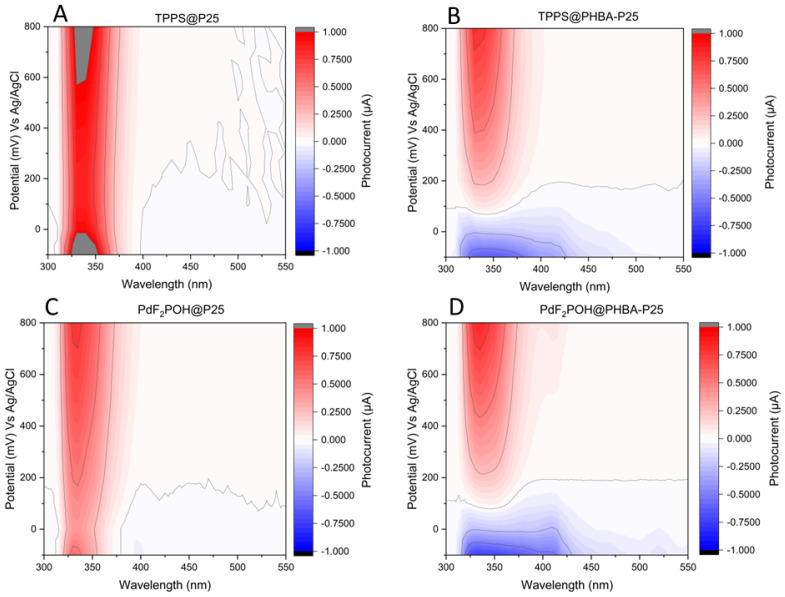
Photocurrent as a function of potential (vs. Ag/AgCl) and incident light wavelength, recorded on ITO electrodes in a 0.1 M KNO_3_ aqueous solution electrolyte (pH = 7). Photocurrent measure for (**A**) TPPS@P25, (**B**) TPPS@PHBA-P25, (**C**) PdF_2_POH@P25, (**D**) PdF_2_POH@PHBA-P25.

**Figure 5:**
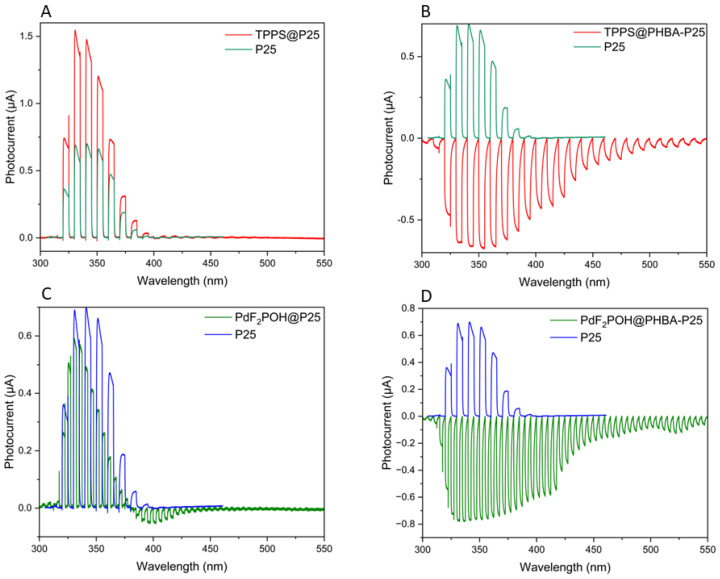
Photocurrents generated by the electrodes covered with P25 and PHBA-P25 with adsorbed (metallo)porphyrins ((**A**)—TPPA@P25, (**B**)—TPPS@PHBA-P25, (**C**)—PdF_2_POH@P25, (**D**)—PdF_2_POH@PHBA-P25) as a function of the wavelength of incident light (applied potential −200 mV vs. Ag/AgCl).

The detection of photocurrents above 400 nm for PdF_2_POH@P25, PdF_2_POH@PHBA-P25, and TPPS@PHBA-P25 confirms that porphyrins sensitize titania to visible light (Figure 4 and Figure 5). Figure 5 presents the photocurrent response to chopping light irradiation. In the case of strongly adsorbed PdF_2_POH@P25, the photocurrents coincide precisely with the energy levels of the Soret and Q_xy_ absorption bands of the porphyrin adsorbed on the TiO_2_ surface. This suggests that both S_0_→S_2_ and S_0_→S_1_ excitations may contribute to the enhanced photochemical activity of the modified titania. In the case of TPPS@P25, there was negligible to no change in photocurrents above 400 nm (Figure 4A and Figure 5A), suggesting that this modification failed to sensitize P25 to visible light. However, in the UV range (325–400 nm), the intensity of the photocurrent was enhanced in comparison to unmodified P25. Comparing the photocurrent measurement results, it becomes obvious that porphyrins sensitize PHBA-P25 material more effectively than P25, and the cathodic current is generated (Scheme 1). The generation of cathodic photocurrent is a consequence of the oxidation of the dye by the produced species [66]. Previous studies indicate that the lower potential oxidation wave can be attributed to the oxidation of the -SO_3_H group. The second oxidation wave, potentially consisting of two unresolved peaks, may be associated with the oxidation of the macrocycle. The assigned oxidation potentials are much lower than the valence band edge potential of titanium, which is about 2.7 V at pH = 7 [67]. This suggests that electron transfer from the valence band of TiO_2_ to the excited dye molecule is unlikely. Given the redox properties of the adsorbed (metallo)porphyrins and P25, the observed visible light-induced photocurrents may be due to electron transfer from the excited dyes to the TiO_2_ conduction band.

Tabari et al. recorded the generation of photocurrent when surface-modified TiO_2_ remained unchanged under different polarization conditions, whether negative or positive ones [68]. Upon UV irradiation in the studied potential range, pristine TiO_2_ generates anodic photocurrents. In the case of unmodified TiO_2_ (Scheme 1a), even under negative polarity, electrons move towards the conductor, since trapping by the redox couple of the electrolyte (e.g., O_2_) proves ineffective. In contrast, for dye-modified TiO_2_, additional pathways (as shown in Scheme 1b–d) elucidate the visible light-induced electrochemical behavior. Under negative polarity conditions, excitation by ultraviolet and visible light led to the observation of photocathode currents as electrons moved from the conductor to the oxidized ground state of dye (HOMO).

### 2.5. Detection of Reactive Oxygen Species in Heterogeneous System

**Figure 6:**
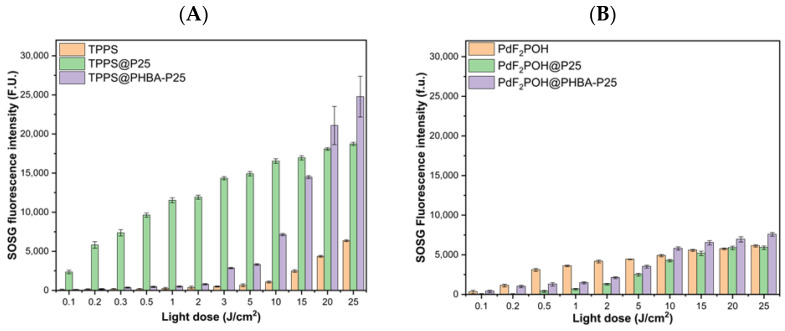
Photogeneration of singlet oxygen. Light dose-dependent increase in fluorescence generated by fluorescent probe—SOSG in the presence of TPPS-based materials (**A**) and PdF_2_POH-based materials (**B**) irradiated with 420 ± 20 nm.

**Figure 7:**
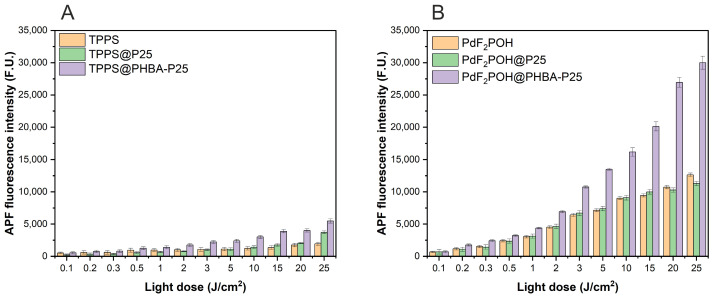
Photogeneration of hydroxyl radicals. Light dose-dependent increase in fluorescence generated by the fluorescein (product of APF reaction with hydroxyl radical) in the presence of TPPS-based materials (**A**) and PdF_2_POH-based materials (**B**) irradiated with 420 ± 20 nm.

One of the most important features of photosensitizers used in PDT is their ability to generate singlet oxygen (type II photochemical reactions) and other ROS (type I photochemical reactions). To assess whether the adsorption of porphyrins on the TiO_2_ surface elevated the generation of singlet oxygen (^1^O_2_), we used the Singlet Oxygen Sensor Green (SOSG) fluorescent probe (Figure 6). For the hydroxyl radical, we used 3′-(*p*-aminophenyl) fluorescein (APF) (Figure 7). Materials were suspended in PBS in concentrations corresponding to absorbance values of 1. The probes were added to the material suspension with a final concentration of probe 50 μM. Prepared solutions were irradiated with LED light at wavelength λ = 420 ± 20 nm. For TPPS@P25 and TPPS@PHBA-P25, the results indicate a much higher quantum yield of singlet oxygen generation compared to TPPS in the homogenous system. At low light doses (0.1–15 J/cm^2^), TPPS@P25 generates singlet oxygen more efficiently; however, at light doses of 20–30 J/cm^2^, TPPS@PHBA-P25 has a higher quantum yield of singlet oxygen generation (Figure 6A). After incorporating the palladium ion into the porphyrin ligand, the mechanism of ROS generation changes in favor of the Type I photochemical reaction mechanism. However, in the case of the palladium derivative, such a large change in the degree of singlet oxygen generation is not observed. On the contrary, at light doses of 0.1–5 J/cm^2^, unmodified porphyrin generates singlet oxygen to a greater extent than when applied to TiO_2_ or TiO_2_ with PHBA. At higher light doses, both materials as well as unmodified porphyrins generate singlet oxygen to a similar extent, suggesting that the application of PdF_2_POH to the composite material does not enhance the generation of ^1^O_2_ (Figure 6B).

For hydroxyl radical generation, the results show an inverse relationship. In the case of unmodified TPPS, hydroxyl radical generation was relatively low for all light doses used. The utilization of TiO_2_-based material and PHBA enhanced type I mechanism reactions only slightly (Figure 7A). The unmodified palladium derivative generates HO· at a higher degree than singlet oxygen, especially at higher light doses (5–30 J/cm^2^). PdF_2_POH deposition has virtually no effect on hydroxyl radical generation efficiency, but the addition of PHBA significantly increases their production. Differences become apparent at light doses higher than 2 J/cm^2^. At a light dose of 30 J/cm^2^, an almost three times higher signal was achieved for PdF_2_POH@PHBA-P25 than for palladium porphyrin alone or porphyrin deposited on unmodified P25 (Figure 7B).

The increased fluorescence of fluorescein, which is a product of the APF reaction with hydroxyl radical for PdF_2_POH@PHBA-P25, and the small fluorescence signal of the SOSG probe suggest that this material enhances the generation of reactive oxygen species by mechanism I photochemical reactions. Nosaka et al. proposed a mechanism for TiO_2_ systems in which the reverse electron transfer occurs from a superoxide ion formed after the one-electron reduction in dioxygen by e_CB_^−^ to the valence band hole or a hole trapped on the TiO_2_ surface [70]. This mechanism was suggested because, under these irradiation conditions, the efficient production of holes is less probable. For photocatalytic purposes, the most optimal would be if the materials generated ROS by two photochemical reactions—^1^O_2_ and OH. Furthermore, it would be beneficial to measure other ROS, such as H_2_O_2_, and O_2_^·−^ to obtain a broad image of the photocatalytic reaction for the hybrid materials.

### 2.6. Photodegradation Studies

**Figure 8:**
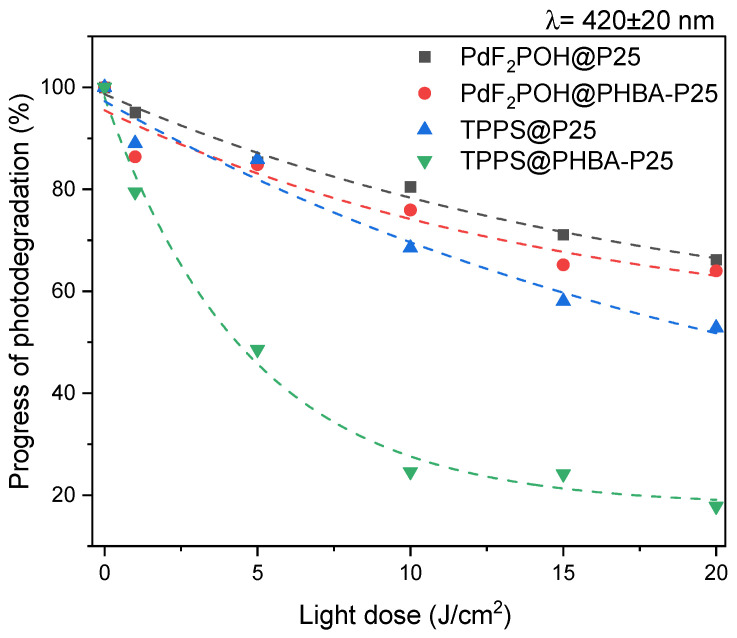
Photodegradation of TiO_2_-modified materials (TPPS@P25, TPPS@PHBA-P25, PdF_2_POH@P25, and PdF_2_POH@PHBA-P25 upon irradiation with visible light (>400 nm).

In environmental applications, it is important for the materials to be sufficiently photostable. Photostability tests were conducted for solid TPPS@P25, TPPS@PHBA-P25, PdF_2_POH@P25, and PdF_2_POH@PHBA-P25 materials mixed with barium sulfate (BaSO_4_) in a ratio of 1:10. Diffuse reflectance spectra of the materials were recorded after the irradiation (λ = 420 ± 20 nm) with the described light doses. The spectra were captured both prior to and immediately after the irradiation, with a time window of 10 to 120 min. Changes in the absorption spectra upon irradiation at 420 nm showed their reduced photochemical stability (Figure 8). The photostability of TPPS@P25, PdF_2_POH@P25, and PdF_2_POH@PHBA-P25 was quite similar. However, palladium-containing materials exhibited the greatest stability. The addition of PHBA to the TPPS-based hybrid material decreased its stability. These results can be connected to the high generation of singlet oxygen by TPPS@PHBA-P25 (Figure 6A). After irradiation with light doses over 5 J/cm^2^, this porphyrin generates a significant amount of singlet oxygen, which probably oxidizes the material, decreasing its photostability.

### 2.7. Photocatalytic Degradation of Methylene Blue and Tramadol Hydrochloride in a Heterogenous System

**Figure 9:**
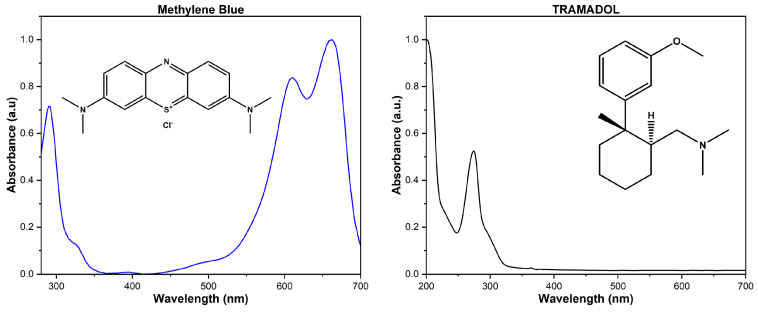
Electronic absorption spectra and chemical structures of two model pollutants—methylene blue (**left**) and tramadol (**right**).

Several researchers investigated the photocatalytic performance of hybrid (metallo)porphyrin-TiO_2_ materials and their adaptable capabilities in electron or energy transfer reactions when exposed to visible light. These materials were examined using model pollutant molecules (such as methyl orange and 4-nitrophenol) and pharmaceutical compounds. The photocatalytic activity of our porphyrin@TiO_2_ hybrid materials was examined towards methylene blue dye (MB) and tramadol hydrochloride (TRML) degradation. The experiments have been performed using continuous white light emitted by an Xe lamp (XBO-150) equipped with a 400 nm cut-on filter for both compounds and, additionally, a 505 nm cut-off filter for MB. Our porphyrin-based materials are perfect for photocatalytic degradation of MB as they absorb in the range of 350–500 nm; therefore, it is possible to avoid the absorption of this model pollutant. The electronic absorption spectra of these two model pollutants, along with their chemical structure, are presented below (Figure 9).

**Figure 10:**
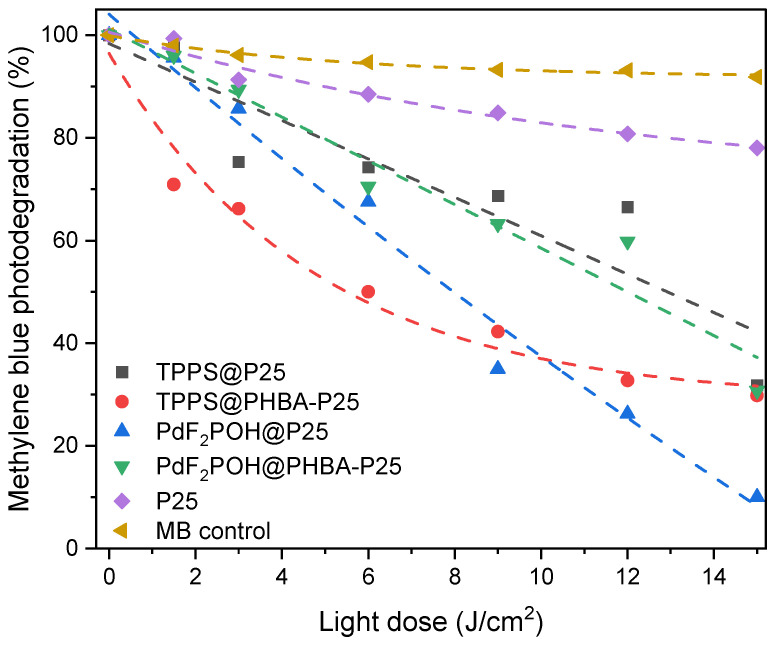
Photodegradation of methylene blue (MB) in presence of various TiO_2_-modified materials: TPPS-based (TPPS@P25, TPPS@PHBA-P25), PdF_2_POH-based (PdF_2_POH@P25, PdF_2_POH@PHBA-P25), unmodified P25, and methylene blue as a control upon irradiation with visible light (with cut-on filter > 400 nm and cut-off filter < 520 nm).

**Figure 11:**
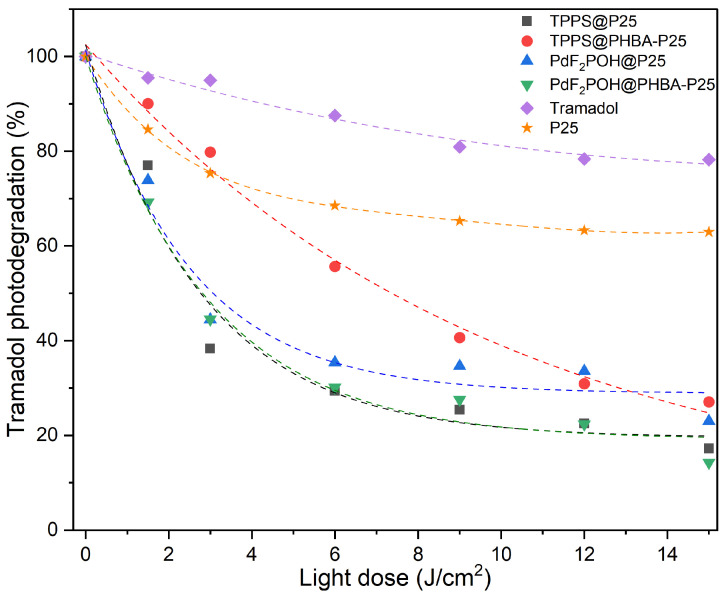
Photodegradation of tramadol (TRML) in the presence of various materials such as TPPS-based (TPPS@P25, TPPS@PHBA-P25), PdF_2_POH-based (PdF_2_POH@P25, PdF_2_POH@PHBA-P25), unmodified P25, and tramadol as a control upon irradiation with visible light (with cut-off filter > 400 nm).

Results of MB photodegradation tests suggest better photocatalytic activity for PdF_2_POH@P25, than for the rest of the tested materials (Figure 10). In the case of PdF_2_POH@P25, we observed almost complete degradation of MB. It is also probable that PdF_2_POH@PHBA-P25 would catalyze full degradation of MB with higher light doses than used in the experiment, similar to TPPS@P25. In the case of TRML, TPPS@P25, and PdF_2_POH@PHBA-P25 exhibited similar activity, showing almost 70% of TRML degradation after irradiation with a light dose of 6 J/cm^2^ (Figure 11). Modification of TPPS-based material showed improvement in photocatalytic activity after incorporation of PHBA into the nanoparticle structure, resulting in almost 90% degradation of MB; however, such a result was not observed for TRML photodegradation [71]. For the latter, TPPS@P25 degraded TRML at 60% after irradiation with a 3 J/cm^2^ light dose. The addition of PHBA reduced the degradation of TRML to 20% after the same dose of light. On the other hand, the addition of PHBA increased the photocatalytic activity of PdF_2_POH-based materials. TPPS@PHBA-P25 exhibited good photocatalytic activity towards MB degradation at lower light doses (1–8 J/cm^2^), but in the end, showed similar activity to the rest of the materials. For TRML degradation, the final result was similar for all tested materials, reaching the degradation of 70–80% of the compound [72]. The results of the tramadol photodegradation tests suggest better photocatalytic activity than the materials we tested previously (F_2_PMet@TiO_2_, ZnF_2_Pmet@TiO_2_, and CoF_2_Pmet@TiO_2_- as described earlier by some of us) [73].

MB alone has shown little photodegradation under visible light; however, with TiO_2_, it was degraded by 80% after irradiation with a light dose of 15 J/cm^2^. TRML alone showed a slight photodegradation at light doses over 6 J/cm^2^, which was enhanced by TiO_2_ catalysis.

## 3. Discussion and Conclusions

Inorganic semiconductors present numerous futures for their applications in environmental, biological, and medical fields. TiO_2_ has garnered particular interest due to its distinctive attributes, including its classification as a wide-band gap semiconductor, biocompatibility with living systems, water stability, and pronounced photocatalytic efficacy. Significantly, TiO_2_ also allows for surface modification through various inorganic and organic molecules, such as tetrapyrrolic and tetraindolic compounds, facilitating the creation of hybrid composites capable of expanding the photoactivity of TiO_2_ to encompass the visible light spectrum. One such modification can be the adsorption of porphyrins widely used in photodynamic therapy and the photodynamic inactivation of microorganisms. The TiO_2_ surface can also be modified with anchoring compounds such as 4-hydroxybenzoic acid (PHBA), which increases the number of porphyrin molecules binding to the surface of nanoparticles.

In our work, we investigated the photochemical properties of newly synthesized porphyrin-based materials—with commercially available porphyrin—TPPS and synthesized by us, never studied before metalloporphyrin—PdF_2_POH. We used a novel approach in the preparation of porphyrin-based composites by impregnation with 4-hydroxybenzoic acid (PHBA), which serves as a linker for higher adsorption on the TiO_2_ surface. Electronic absorption spectra and DLS measurements (size and ζ-potential) prove the effective adsorption of all tested porphyrins on the P25 surface. We managed to successfully sensitize TiO_2_ with three of the four prepared materials, which is proven by photoelectrochemical measurements. Our research also proved that the adsorption of porphyrins on the surface of TiO_2_ modified the generation of ROS, mainly singlet oxygen for TPPS-based materials and hydroxyl radical for PdF_2_POH-based materials. Composites with the addition of PHBA showed higher quantum yields of both singlet oxygen and hydroxyl radicals. The adsorption of TPPS on the P25 surface enhanced the generation of ^1^O_2_ but did not cause drastic changes in hydroxyl radical generation. Opposite results were obtained for PdF_2_POH—metalloporphyrin-based materials did not influence singlet oxygen generation but increased the production of hydroxyl radicals. The inherent capacity of nanomaterials to facilitate electron transfer, thereby promoting ROS generation, constitutes an intrinsic characteristic of these materials and ensures their effective application as a photocatalyst for multiple purposes. Our findings demonstrate that the surface modification of P25 resulted in the generation of singlet oxygen and hydroxyl radicals, correlating with the physicochemical attributes concerning mechanisms and activity. Moreover, PdF_2_POH leads to the generation of a higher amount of ROS compared to previously synthesized F_2_POH@P25 and ZnF_2_POH@P25. The incorporation of PHBA into the structure of the composite enhanced the generation of singlet oxygen for TPPS-based material and hydroxyl radical for PdF_2_POH-based material.

Palladium (II) porphyrin-based materials—PdF_2_POH@P25 and PdF_2_POH@PHBA-P25—were more photostable than materials synthesized before in our group [49]. Low TPPS@PHBA-P25 photostability can be caused by its high singlet oxygen generation, which can lead to the oxidation of the material and its low stability after irradiation with higher light doses. Therefore, when designing hybrid composites, it is important to balance ROS generation with photostability. High generation of singlet oxygen can lead to oxidation of the material, which limits its photostability and photocatalytic activity. PdF_2_POH@P25 managed to catalyze the photodegradation process almost to completion. PdF_2_POH@PHBA-P25 also has the potential to fully catalyze the degradation process, but higher light doses are required. In the case of TRML degradation, all hybrid materials degraded the model drug pollutant by 70–80%. The best results were obtained for TPPS@P25, PdF_2_POH@P25, and PdF_2_POH@PHBA-P25 materials, which catalyze photodegradation by 50% after irradiation with a 3 J/cm^2^ light dose. TPPS@PHBA-P25 catalyzed the degradation of TRML slower in comparison to other synthesized materials.

Collectively, the comprehensive investigations conducted in this study affirm that TiO_2_ nanoparticles, when modified with porphyrins and metalloporphyrins, exhibit photoactivity in the visible light-range, enhanced ROS generation, and potential in visible light-driven photocatalysis. Moreover, all synthesized compounds and materials also have a great promise for the photodynamic inactivation of microorganisms, so further research into their biological activity is currently being undertaken.

## 4. Materials and Methods

### 4.1. Physicochemical Properties

#### 4.1.1. Preparation of PHBA-P25 Materials

Degussa P25 TiO_2_ (1200 mg) and a solution of PHBA (100 mg) in deionized water (20 mL) were mixed and placed in the ultrasonic bath for 1 h. The resulting homogenous mixture was protected from light and shaken overnight. After centrifugation (9000 RPM, 15 min), the solid residue was washed with deionized water (20 mL) and centrifugated again. The bulky yellowish residue was dried at 80 °C for 12 h. After drying, the solid was ground in the agate mortar to obtain PHBA-P25 as a yellow powder. This protocol is a modified version of the protocol from Mahy et al. [60].

#### 4.1.2. Preparation of TPPS/PdF_2_POH Hybrid Nanomaterials

Here, 500 µM porphyrin solution in DMF (2 mL) and a solution of appropriate TiO_2_ nanomaterial (200 mg) in Tris buffer (2 mL) was mixed and placed in the ultrasonic bath at 60 °C for 2 h. The resulting pinkish suspension was protected from light and shaken overnight. After centrifugation (9000 RPM, 15 min), the solid residue was washed with deionized water (5 mL) and centrifugated again. The bulky pale pink residue was dried at 60 °C for 12 h. After drying, the solid was ground in the agate mortar to obtain material powder.

#### 4.1.3. UV-Vis-NIR Electronic Absorption Spectra Measurements

The photosensitizer samples were dissolved in Tris buffer, pH 7.4. UV-Vis absorption spectra were recorded in quartz cuvettes (l = 1 cm) with a UV-3600 Shimadzu UV-Vis-NIR spectrophotometer (Shimadzu, Kyoto, Japan) in the range of 350–700 nm.

#### 4.1.4. Dynamic Light Scattering (DLS)

ζ-potential and hydrodynamic diameter measurements of nanomaterials in colloid materials were performed using Zetasizer Ultra ZS (Malvern Instruments, Malvern, UK). The apparent diffusion coefficients of the nanoparticles were obtained from the normalized time correlation function of the scattered electric field, g(1) (τ), using the cumulants analysis. An average value was obtained from repeated measurements for each sample (N = 5).

#### 4.1.5. Detection of Reactive Oxygen Species Using Fluorescent Probes

3′-(*p*-aminophenyl) fluorescein (APF) and singlet oxygen sensor green (SOSG) probes were employed for the detection of ROS generated during illumination of the colloid materials and photosensitizer itself. The compound absorbance was at a level of about 1.0. Each fluorescent probe was added to a well at a final concentration of 50 µM. Freshly prepared solutions were then irradiated with LED (420 ± 20 nm) light for increasing time intervals. A Tecan Infinite M200 Reader microplate reader was employed to capture the fluorescence signal both immediately before and right after exposure to light. When APF was utilized, a fluorescence signal at 515 nm was detected after excitation at 490 nm. In the case of SOSG, fluorescence emission was observed at 525 nm, with an excitation wavelength of 505 nm.

#### 4.1.6. Photoelectrochemical Measurements

Photocurrents of modified and unmodified TiO_2_ were measured using a three-electrode setup with a 0.1 M KNO_3_ electrolyte. The working electrode was prepared by spreading the studied material on an ITO-coated transparent foil and drying it with warm air. A platinum wire and Ag/AgCl (3 M) were used as the counter and reference electrodes, respectively. Photoelectrochemical experiments were conducted utilizing a consistent potentiostat and a 150 W xenon lamp (Photon Institute, Varanasi, India), featuring an automated monochromator with a shutter (Photon Institute) employed as the illumination source, emitting monochromatic light spanning the entire UV and visible light spectrum. The working electrodes were exposed to irradiation from the rear surface.

#### 4.1.7. Photodegradation Studies

Photodegradation of hybrid materials was monitored by the measurements of the changes in the diffuse reflectance spectra recorded for the materials irradiated with 420 ± 20 nm LED light. The DRS spectra were recorded before and immediately after irradiation with light doses from 1 to 20 J/cm^2^ using a Shimadzu (Kyoto, Japan) 3600 UV-Vis-NIR spectrophotometer.

#### 4.1.8. Photocatalytic Activity of the Hybrid Materials

The photocatalytic activity of porphyrin@TiO_2_ materials was tested by monitoring the progress of tramadol hydrochloride (TRML) and methylene blue (MB) (both purchased from Sigma-Aldrich, Burlington, MA, USA) photodegradation in Tris solution. Continuous irradiation of a suspension of TiO_2_ impregnated with porphyrins (7 mg) in solution of model pollutants (14 mL of both: B: c_0_ = 6.71 × 10^−4^ M, TRML: c_0_ = 17 mg/L) was carried out using the xenon lamp (XBO-150) equipped with the copper (II) sulfate filter (10 cm optical length) and a 400 cut-on (and 505 nm cut-off filter for MB), delivering 1.7 mW/cm^2^. The suspension was stirred and irradiated in a cylindrical quartz cuvette (l = 1 cm). Application of this filter enabled the excitation of hybrid materials in the visible range of electromagnetic radiation without the excitation of neither TRML nor MB. The progress of the reactions indicating pollutant degradation was monitored by the measurements of the electronic absorption spectra of TRML and MB. A UV-3600 Shimadzu UV-Vis-NIR spectrophotometer was used for all of these experiments.

## Figures and Tables

**Table 1:** Particle size and ζ-potential of the tested nanomaterials.

Material	Size/nm DLS	ζ/mV
P25	146	21
TPPS@P25	154	−9
TPPS@PHBA-P25	155	−33
PdF_2_POH@P25	170	22
PdF_2_POH@PHBA-P25	197	12

## Data Availability

Data are contained within the article.

## Abbreviations

AOP	Advanced oxidation processes
APF	3′-(p-aminophenyl) fluorescein
CO_2_	Carbon dioxide
DLS	Dynamic Light Scattering
DMF	Dimethylformamide
DNA	Deoxyribonucleic acid
DRS	Diffuse reflectance spectroscopy
e_CB_^−^	Relative conduction band
H_2_O_2_	Hydrogen peroxide
ISC	Intersystem crossing
ITO	Indium Tin Oxide
KNO_3_	Potassium nitrate
MB	Methylene blue
OH·	Hydroxyl radicals
^1^O_2_	Singlet oxygen
TPPS	5,10,15,20-tetrakis(4-sulfophenyl)porphyrin
TiO_2_	Titanium(IV) oxide
P25	Type of Titanium(IV) oxide, Anatase-Rutile ratio: 85:15
PBS	Phosphate buffered saline
PdF_2_POH	Palladium(II) 5,10,15,20-tetrakis(2,6-difluoro-3-sulfophenyl)porphyrin
PDI	Photodynamic inactivation
PHBA	4-hydroxybenzoic acid
ROS	reactive oxygen species
SOSG	Singlet Oxygen Sensor Green
TRML	Tramadol
